# Nucleic acid extraction from formalin-fixed paraffin-embedded cancer cell line samples: a trade off between quantity and quality?

**DOI:** 10.1186/s12907-016-0039-3

**Published:** 2016-11-14

**Authors:** Caroline Seiler, Alan Sharpe, J. Carl Barrett, Elizabeth A. Harrington, Emma V. Jones, Gayle B. Marshall

**Affiliations:** 1 AstraZeneca Oncology Innovative Medicines, Alderley Park, Macclesfield, UK; 2 AstraZeneca, Cambridge, UK; 3 AstraZeneca, Waltham, USA; 4 Leeds University-AZ Sandwich Placement, Leeds, UK; 5 AstraZeneca, 8AF6, Mereside, Alderley Park, Alderley Edge, Cheshire SK10 4TG UK

**Keywords:** FFPE, DNA, RNA, Manual, Automated, Extraction

## Abstract

**Background:**

Advanced genomic techniques such as Next-Generation-Sequencing (NGS) and gene expression profiling, including NanoString, are vital for the development of personalised medicines, as they enable molecular disease classification. This has become increasingly important in the treatment of cancer, aiding patient selection. However, it requires efficient nucleic acid extraction often from formalin-fixed paraffin-embedded tissue (FFPE).

**Methods:**

Here we provide a comparison of several commercially available manual and automated methods for DNA and/or RNA extraction from FFPE cancer cell line samples from Qiagen, life Technologies and Promega. Differing extraction geometric mean yields were evaluated across each of the kits tested, assessing dual DNA/RNA extraction vs. specialised single extraction, manual silica column based extraction techniques vs. automated magnetic bead based methods along with a comparison of subsequent nucleic acid purity methods, providing a full evaluation of nucleic acids isolated.

**Results:**

Out of the four RNA extraction kits evaluated the RNeasy FFPE kit, from Qiagen, gave superior geometric mean yields, whilst the Maxwell 16 automated method, from Promega, yielded the highest quality RNA by quantitative real time RT-PCR. Of the DNA extraction kits evaluated the PicoPure DNA kit, from Life Technologies, isolated 2–14× more DNA. A miniaturised qPCR assay was developed for DNA quantification and quality assessment.

**Conclusions:**

Careful consideration of an extraction kit is necessary dependent on quality or quantity of material required. Here we provide a flow diagram on the factors to consider when choosing an extraction kit as well as how to accurately quantify and QC the extracted material.

**Electronic supplementary material:**

The online version of this article (doi:10.1186/s12907-016-0039-3) contains supplementary material, which is available to authorized users.

## Background

Highly multiplexed assays, capable of profiling many genetic biomarkers in a single experiment, are of rising importance in the field of life sciences, enabling mapping of entire biological pathways [1–5]. Our rapidly growing understanding of the molecular mechanisms driving disease progression enables acceleration of personalised medicines into the clinic, particularly in oncology [6–8]. Such assays require sufficient quantities of high quality DNA and RNA extracted from clinically relevant patient samples.

Formalin is the most widely used fixative, used for over a century by hospitals to preserve clinical samples for long term storage. Extensive collections of formalin fixed paraffin embedded (FFPE) clinical samples exist worldwide, representing an invaluable resource for prospective and retrospective studies on archival tissue [9, 10]. However, multiple factors influence the efficiency of formalin fixation including tissue size, fixation temperature and duration, and the amount of time that elapses before the sample is fixed [11]. The lack of standardised procedures for collecting and processing tissue samples results in a range of FFPE qualities across different sites which can be further influenced by the age of the tissue block [10]. Scientists are constantly faced with the challenge of obtaining sufficient amounts of high quality nucleic acids from sub-optimal FFPE samples containing minimal amounts of tissue. Furthermore, the fixation process leads to the cross linking of nucleic acids and proteins resulting in highly fragmented nucleic acid species with amplimers around 100 bases. This can introduce sequence alterations and mono methylol addition of nucleic acids which can impact downstream PCR based assays [12–15]. A number of companies supply off the shelf kits optimised for the extraction of DNA and/or RNA from FFPE sections using varying amounts of tissue. Therefore, we carefully selected seven commercially available FFPE extraction kits with a broad range of properties to assess nucleic acid yield and the quality and purity of the extracted material.

Differing extraction methods were assessed: dual DNA/RNA extraction vs. specialised single extraction, manual silica column based extraction techniques vs. automated magnetic bead based methods and finally a comparison between extraction kits across a range of manufacturers. A number of papers have been published previously comparing the performance of manual FFPE extractions kits from different suppliers [16–18], with more recent studies focusing on comparisons between automated extraction methods and their manual counterpart in order to reduce the hands on time of laborious extraction processes [19–22]. However, the studies use different samples and methods for quantifying and assessing the quality of the extracted material that are not always comparable, making it challenging to cross compare disparate data sets. It is for this reason that we present a comprehensive analysis of seven selected kits using a single sample set. In addition, we provide a head-to-head comparison of commonly used platforms to aid decisions around how to extract, quantify, and assess the quality of nucleic acids.

Cell blocks were used to ensure sufficient sections could be generated to enable robust analysis of each extraction method; although tissue blocks were initially used, the material was rapidly exhausted thereby preventing all extraction kits from being evaluated across all variables using tissue from a single block. Cancer cell lines were cultured, fixed in formalin and embedded in paraffin, to represent the process of preserving clinical tissue whilst achieving a homogenous cell population with known expression of genes of interest for downstream evaluation. This ensured that variability in this assessment is solely due to differences in the extraction kits and not by fixation methods, times and heterogeneity of the tissue. An additional advantage of having a large quantity of cells embedded within a block, is that it allows each kit to be tested using optimal conditions, so significant differences between kits can be identified.

## Methods

### Formalin fixed paraffin embedded cancer cell line blocks

Human chronic myeloid leukaemia and breast cancer cell lines, used in this study, were obtained from the American Type Culture Collection (ATCC) and the Deutsche Sammlung von Mikroorganismen und Zellkulturen (DSMZ). Cells were grown in T225 tissue culture flasks in RPMI1640 media (phenol red free; Sigma) supplemented with 10% foetal calf serum and 1% L-Glutamine (Life Technologies Bethesda Research Laboratories Ltd) and maintained at 37 °C with 5% CO_2_. Each cell line was passaged up to 3 times and at approximately 80% confluence, the cells were fixed in 10% neutral buffered formalin for 24 h at room temperature before embedding in paraffin wax. Consecutive 5 μm sections were generated from each of the FFPE cell blocks using a standard microtome blade and fixed onto glass microscope slides. All FFPE cell blocks and 5 μm sections were stored at room temperature until nucleic acid extraction was performed.

### Nucleic acid extraction

Seven commercially available nucleic acid extraction kits were used in this study (Table 1). Kits were selected based on prior reviews of performance [21, 23] and their extraction properties. Four of the kits tested, rely on the selective binding of nucleic acids to silica columns, two of the kits exploit binding to paramagnetic beads allowing automation of the extraction and one kit tested did not require nucleic acid binding to a solid interphase. Another factor taken into account when selecting kits was the suitability of the extracted material for use in downstream applications including RT-qPCR, qPCR, and gene expression analysis.

**Table 1:** Summary of the seven off the shelf nucleic extraction kits evaluated in this study, including the key differences between each method

Extraction kit	Manufacturer	Extracted Material	Input tissue amount	Elution Volume	Purification Method	Level of automation	Proteinase K digestion	DNase I digestion	Geometric mean Yield (ng)
RNeasy FFPE	Qiagen	RNA	Up to 40 μm	30 μl	Silica Column	Manual	15 min at 56 °C, 15 min at 80 °C	15 min at room temperature in the cell lysate	398.0
Arcturus Paradise plus RNA extraction and isolation	Life technologies	RNA	Up to 40 μm	12 μl	Silica Column	Manual	5 h at 37 °C	20 min at room temperature on the column	197.0
Maxwell 16 LEV RNA FFPE Kit	Promega	RNA	Up to 10 μm	50 μl	Paramagnetic beads	Automated	15 min at 56 °C, 60 min at 80 °C	15 min at room temperature in the cell lysate	231.5
AllPrep DNA/RNA FFPE	Qiagen	RNA & DNA	Up to 40 μm	RNA 30 μl DNA 50 μl	Silica Column	Manual	15 min at 56 °C	15 min at room temperature on the column	RNA 267.7 DNA 10.1
Arcturus PicoPure DNA extraction kit	Life technologies	DNA	1.5–2.0 μg tissue	150 μl	Single tube extraction	Manual	24 h at 65 °C (lyophilised before use)	N/A	172.6
QIAmp DNA FFPE tissue	Qiagen	DNA	Up to 80 μm	50 μl	Silica Column	Manual	60 min at 56 °C, 60 min at 80 °C	N/A	40.9
Maxwell 16 FFPE Tissue LEV DNA Purification Kit	Promega	DNA	Up to 50 μm	50 μl	Paramagnetic beads	Automated	Overnight at 70 °C	N/A	88.3

A total of 30 samples from 6 FFPE cell line blocks were generated using each extraction method yielding a total of 240 samples as one method extracted both DNA and RNA. RNA extractions were performed on the following cell line blocks: KCL22, MDA-MB-468, MDA-MB-453 (fixed less than 6 months prior to this work) and MDA-MB-231, MDA-MB-468 and MDA-MB-453 (fixed 2–3 years prior to this work). DNA extractions were performed on the following cell line blocks T47D, MDA-MB-468 and MDA-MB-453 from each age group (Table 2). 15 consecutive 5 μm sections were generated from each block and processed as 5 μm, 10 μm (2 combined sections), 15 μm (3 combined sections), 20 μm (4 combined sections) and 25 μm (5 combined sections) in each extraction method. All FFPE sections were deparaffinised using an automated protocol on the Leica autostainer XL, involving immersion into xylene twice followed by immersion into 100% ethanol twice then left to air dry for 15 min, with the exception of the two Promega kits where no deparaffinisation was performed. All extraction kits were used according to the manufacturer’s instructions including elution volumes quoted and all optional DNase 1 steps in RNA extractions. Following deparaffinisation, the surface area of the cell line pellet was checked by eye from consecutive sections within a block to ensure they matched. All RNA samples were stored at −80 °C and all DNA and cDNA samples were stored at −20 °C throughout the study.

**Table 2:** Summary of the FFPE cell pellet blocks used in the study

	<6 months of age	>2 years of age
RNA extraction kits	KCL22, MDA-MB-468, MDA-MB-453	MDA-MB-231, MDA-MB-468, MDA-MB-453
DNA extraction kits	T47D, MDA-MB-453, MDA-MB-468	T47D, MDA-MB-453, MDA-MB-468

### Quantitative and qualitative RNA assessment

#### Yield

RNA concentration was determined using the Qubit HS RNA assay (Life Technologies, catalogue number Q32852) on the Qubit 2.0 fluorometer. 1.0 μl of RNA in a total 200 μl volume of working solution was prepared. The fluorescence of unknown RNA samples was measured and converted to RNA concentration using a calibration curve generated from newly prepared RNA standards at 0 ng/μl and 10 ng/μl.

#### Integrity

The extent of RNA fragmentation was assessed via capillary chip electrophoresis. RNA samples at a concentration ≥5 ng/μl were tested using RNA 6000 nano LabChips. The chips were run on the Agilent 2100 Bioanalyser according to the manufacturer’s instructions.

#### Purity

RNA concentration and purity were measured on the Nanodrop 2000 using 1 μl of RNA. The ratio between the absorbance at 260 nm and 280 nm was used to evaluate purity; we assumed ratios between 1.8 and 2.0 to be pure.

### mRNA quality assessment

Multiplex quantitative 2-step reverse transcriptase PCR was performed to assess mRNA quality for the same sample set analysed on the Agilent. 7 μl of RNA at a concentration of 5 ng/μl (quantified using the Qubit 2.0 fluorometer) was used to generate cDNA in a 10 μl total extraction volume using the SuperScript VILO cDNA synthesis kit (Life Technologies, Catalogue Number 11754-050) followed by incubation at 25 °C for 10 min, 42 °C for 60 min and 85 °C for 5 min.

The qPCR assay was prepared in Roche 384 well PCR plates (product no. 04729749001) in a 10 μl reaction volume fired using an ECHO 525 non-contact micro dispenser (LabCyte). Three primer probe pairs were used in each assay against the following housekeeping genes: RPLP0 (CY5), RPL19 (FAM) and ACTB (CYAN500) using three unique fluorophores compatible with the Roche LightCycler480 PCR machine. 0.5 μl of cDNA at 3.5 ng/μl (within the linear range of the assay, Additional file 1: Figure S1) was run in duplicate alongside negative controls lacking the cDNA template on the Roche LightCycler480 PCR machine with 1 pre incubation cycle at 95 °C for 10 min followed by 45 amplification cycles at 95 °C for 10 s, 60 °C for 30 s, 72 °C for 1 s before cooling to 40 °C for 30 s. Data was analysed using the LightCycler 480 software (version 1.5.1.62) under the “Abs quant/ 2nd Derivative Max analyses” programme. This software calculated the average Cq per gene for sample duplicates; this was then used to calculate the average across the 3 house keeping genes and this value was used to compare mRNA quality obtained using the different extraction kits, lower Cq values indicated higher quality mRNA.

### Quantitative and qualitative DNA assessment

For DNA to be used in downstream genomic techniques, for example NGS, quality and quantity of amplifiable DNA needs to be assessed. Qubit and Nanodrop will not generate this information so Quantitative PCR was performed for DNA quantification and quality assessment, using the hgDNA Quantification and QC kit from Kapa Biosystems (KK4960) as per the manufacturer’s instructions. Two amplicons from a conserved single copy DNA locus were generated, one 41 bp in length and one 129 bp in length. Amplification of the 41 bp target was used for quantitation of amplifiable DNA. This was compared to data generated using the 129 bp assay, to ensure valid conclusion for the Qiagen extraction kits that are less able to retain fragments <100 bp. The 129 bp assay was used to assess DNA quality, since poor DNA quality influences the ability to amplify longer DNA fragments. The relative quality of DNA samples was calculated by normalising the concentration obtained using the 129 bp assay against the concentration obtained from the 41 bp assay, which generates a Q-ratio value between 0 and 1 (0 represents highly fragmented DNA whilst 1 represents high quality DNA). Both assays were performed on the Roche LightCycler480 PCR machine in a 3.0 μl reaction volume. Data was analysed using the Roche LC480 software using the “Abs quant/ 2nd Derivative Max analyses” programme. This software calculated the average Cq value of duplicates, the standard deviation and DNA concentrations were generated from a standard curve.

### Statistical evaluation

Graphical representations were generated using TIBCO Spotfire software version 5.0 and Microsoft Office Excel 2007. Data was represented as the geometric mean with corresponding geometric standard error as the data was found to have an asymmetrical distribution. The statistical significance of the data was assessed using two sample unequal variance t tests as preliminary analysis was performed in the absence of an appropriate rationale as to the direction of the relationship. In the results and discussion, we refer to P-values <0.05 as significant. The coefficient of variation was calculated for correlation plots to assess the fit of simple linear regressions.

## Results and discussion

### Total RNA yield

**Fig. 1:**
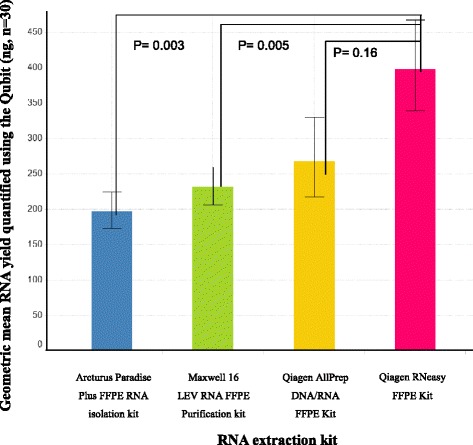
Comparison of Qubit RNA yields obtained using four off the shelf extraction kits: Geometric mean RNA yield and associated geometric standard error of mean for the four extraction kits tested in this study, *n* = 30 for each method. P-values show the statistical significance of the difference in yield between each method

All 4 methods successfully extracted RNA (determined as generating a measurable signal using the Qubit) from as little as one 5 μm section, with geometric mean RNA yields greater than 100 ng (Table 1/Fig. 1). The Qiagen RNeasy FFPE extraction kit gave a superior geometric mean RNA yield of 398 ng; however, the difference in the performance of the RNeasy single RNA extraction and the AllPrep DNA/RNA FFPE kit was not significant (P-value 0.16).

Both Qiagen RNA extraction kits significantly outperformed the Artcturus paradise plus FFPE RNA isolation kit and the automated Maxwell 16 LEV RNA FFPE Kit. The RNeasy FFPE Kit gave geometric mean RNA yields 166 ng greater than the Maxwell 16 and 200 ng greater than the Artcturus paradise plus FFPE RNA isolation kit. The AllPrep gave geometric mean RNA yields 36 ng greater than the Maxwell 16 and 70 ng greater than the Arcturus paradise plus FFPE RNA isolation kit. Multiple factors may contribute to the lower RNA yields obtained using the Artcturus paradise plus FFPE RNA isolation kit. Firstly, the lower temperature used during tissue digestion may less efficiently reverse formaldehyde-induced cross links between nucleic acids and proteins, previous studies have reported heating tissues at higher temperatures (>50 °C) as being more efficient [13, 23]. Also the lengthy 5 h digestion time may not be necessary as previous papers have published that no more than 3 h is required [24]. Finally, this method uses less than half the elution volume of the two Qiagen kits potentially compromising the efficiency of the elution buffer to cover the silica membrane which could contribute to the lower RNA yield observed. Despite the lower yield, the Artcturus kit gave the highest RNA concentration which may be desirable for some studies (Table 1).

The Maxwell 16 RNA extraction is the only method that does not involve deparaffinisation; we hypothesise that excess paraffin may reduce the efficiency of cell lysis, since some studies have shown deparaffinisation to be a key pre-treatment step [25]. In addition, this protocol uses paramagnetic beads for purification as opposed to silica columns. The speed at which the beads are mixed with the cell lysate has been shown to have a strong influence on the efficiency of nucleic acid binding with faster mixing speeds being linked to higher RNA yields. Furthermore, the process of transferring the beads through a series of wash buffers can result in the loss of beads which will negatively impact yield. Although the Maxwell 16 automated the binding, washing, and elution steps, up-front sample preparation was required which took approximately 2 h before loading the lysate onto the machine. In addition, it limited the number of samples processed per run to 16 as opposed to batches of 24, which we feel can comfortably be processed using any of the manual extraction kits. This shows the automated RNA extraction on the Maxwell 16 provides no increase in throughput, and we saw no advantage in terms of yield when using this bead based purification method for RNA extraction over silica columns.

**Fig. 2:**
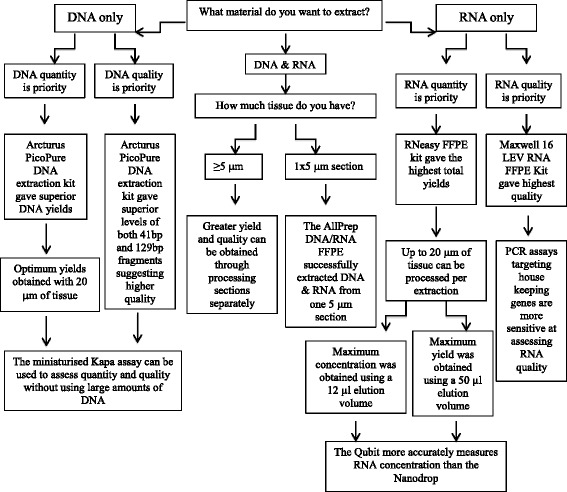
Flow diagram summarising the key points from this study: Decision tree to highlight which extraction kits demonstrated superior performance in terms of nucleic acid yield and quality as well as guidance on how to quantify and QC the extracted material

Based on this data, the Qiagen kits (AllPrep and RNeasy) demonstrated superior performance when mRNA expression and quantity of RNA is the priority. However, if quality is key and quantity of less importance, we would recommend the automated Maxwell 16 LEV FFPE kit (Fig. 2).

### Fluorescence vs. absorbance for RNA quantification

Each of the extracted RNA samples were quantified using both the Qubit RNA HS fluorescence based assay and the Nanodrop 2000 spectrophotometer (for 5 samples there was insufficient sample remaining to run on the Nanodrop). The Nanodrop also generated A260/A280 ratios as a means of assessing purity. All four RNA extraction kits gave mean ratios ≥1.8 indicating good purity.

Nanodrop consistently underestimated the RNA concentration due to the lower sensitivity of the assay (Additional file 2: Figure S2). The limit of detection of the Nanodrop 2000 is 2 ng/μl whereas for the Qubit it is 0.25 ng/μl; 37% of the samples extracted using the Maxwell 16 gave RNA concentrations <2 ng/μl but greater that 0.25 ng/μl and therefore fell within the background noise of the Nanodrop assay but not the Qubit. For these reasons we chose to use RNA concentration readings generated from the Qubit in all subsequent analyses.

### Effect of tissue input on RNA concentration/yield using a fixed elution volume

Total nucleic acid yield and concentration are influenced by the elution volume and the amount of tissue processed. We investigated the effect of varying the amount of tissue on nucleic acid concentration and yield by processing 5 μm, 10 μm, 15 μm, 20 μm and 25 μm of tissue.

**Fig. 3:**
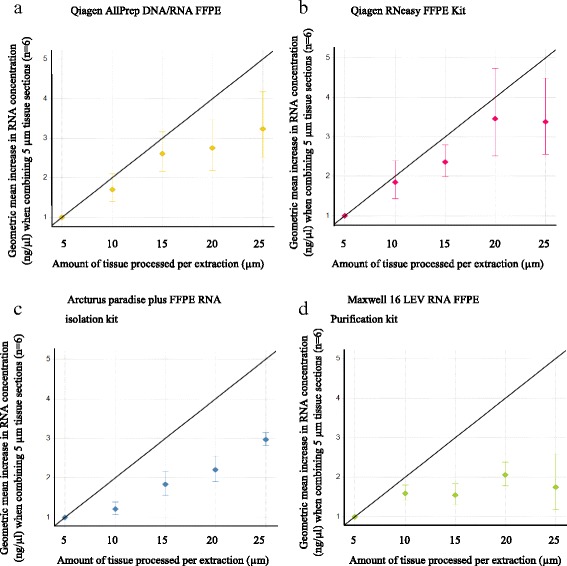
Investigating the relationship between RNA concentration and the input amount of tissue: Geometric mean increase in RNA concentration (*n* = 6) when combining 5 μm sections in a linear fashion and associated standard error of mean, line of Y = X/5 represents a linear relationship **a** Qiagen AllPrep DNA/RNA FFPE kit, **b** Qiagen RNeasy, **c** Arcturus Paradise plus FFPE RNA isolation kit, **d** Maxwell 16 LEV RNA FFPE Purification kit

The Qiagen extraction kits demonstrated a clear linear relationship in concentration with increasing tissue amount (Fig. 3). The Dual extraction kit showed extraction efficiencies of up to 87% (assuming the 5 μm section has 100% extraction efficiency) when processing ≤15 μm of tissue. The RNeasy FFPE kit gave extraction efficiencies of 86% when processing up to 20 μm of tissue, above this the efficiencies were much less. We felt these small losses in yield (~14%) were an acceptable compromise for the increase in concentration achieved through combining tissue sections (Fig. 3a and b). However, the Arcturus Paradise plus RNA extraction kit showed extraction efficiencies between 55 and 60% when processing between 10 and 25 μm of tissue; 45% of the RNA was lost through combining the tissue sections (Fig. 3c). The Maxwell 16 automated RNA extraction method had a very narrow linear range of 5–10 μm tissue. Within this range, 80% extraction efficiency was obtained, but when >10 μm tissue was used a 50% reduction in yield was observed (Fig. 3d).

Therefore, our data shows that combining tissue sections offers no advantage in terms of yield when compared to extracting sections individually, but does provide a method of increasing RNA concentration although in a non-linear fashion.

### Effect of elution volume on RNA concentration/yield using a fixed amount of tissue

In previous experiments the RNeasy FFPE kit gave superior RNA yields, therefore we investigated the relationship between RNA yield/concentration and elution volume. 12 μl, 20 μl, 30 μl 40 μl and 50 μl elution volumes were tested using 5 μm of tissue from four different FFPE cancer cell line blocks.

Results showed a 20 μl elution volume was optimal and resulted in high RNA yields and RNA concentrations (Additional file 3: Figure S3). However, we acknowledge that the amount of RNA bound to the column will also have an impact on elution efficiency; samples with higher amounts of RNA may require larger elution volumes.

### RNA quality assessment: Agilent vs RT-PCR

RNA samples with a concentration ≥5 ng/μl (limit of detection of the Agilent nano assay), obtained using four different extraction kits, were analysed for quality using the Agilent RNA 6000 nano assay. The assigned RNA integrity number (RIN) values were compared to the in house multiplex RT-PCR assay as an assessment of amplifiable RNA. The RNA samples fell into two groups, those obtained from FFPE cell line pellets generated <6 months prior to commencing the study and those obtained from FFPE cell line pellets generated >2 years prior to commencing the study.

**Fig. 4:**
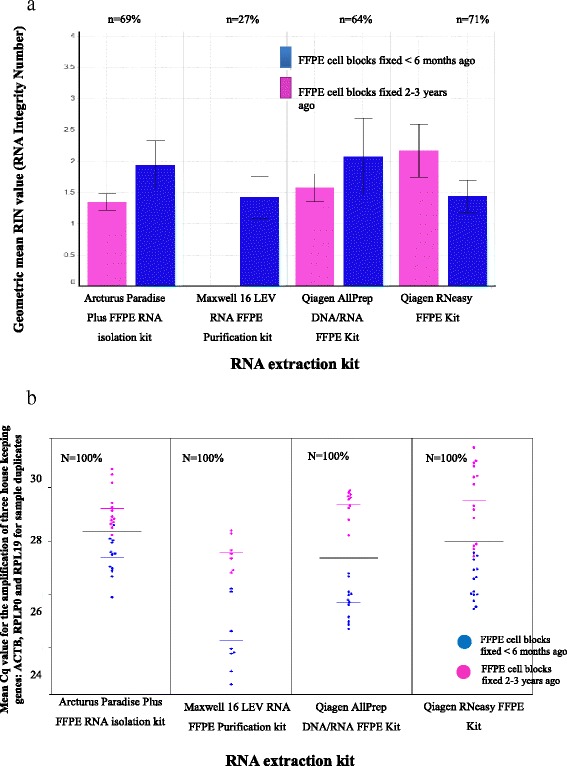
Comparison of the Agilent RNA assay and our in house PCR assay for assessing RNA quality: **a** Agilent RNA assay, graph shows Geometric mean RIN values and associated standard error of mean for RNA samples extracted using four different RNA extraction kits from FFPE blocks <6 months of age or between 2 and 3 years of age. N represents the percentage of samples analysed that were assigned a RIN value. **b** In-house PCR assay, graph shows Mean Cq value for the amplification of three housekeeping genes: ACTB, RPLP0 and RPL19 for sample duplicates, for RNA samples extracted using four different RNA extraction kits from FFPE blocks either <6 months of age (*blue*) or between 2 and 3 years of age (*pink*), horizontal black lines represent the mean Cq for the entire sample population, with pink and blue lines for the two subgroups of FFPE blocks, N represents the percentage of samples analysed assigned a Cq value

The percentage of samples assigned a RIN value varies between the kits tested: 69% of samples were assigned a RIN value for the Arcturus Paradise Plus RNA kit, 13% for the Maxwell 16 LEV RNA FFPE kit, 64% for the AllPrep DNA/RNA FFPE kit and 71% for the Qiagen RNeasy FFPE kit. However, the assigned RIN values failed to show any significant differences in the quality of the RNA released (Fig. 4a).

To allow for a more sensitive, quantitative assessment of RNA quality isolated from FFPE samples, we developed a multiplex PCR assay in house targeting 3 housekeeping genes ActB, RPL19 and RPLP0 to asses RNA quality based on the ability to amplify targets. The multiplex PCR assay showed good concordance with single-plex PCR assays for each housekeeping gene (Additional file 4: Figure S4) and little intra-assay variation was seen, demonstrated by the strong correlation (R^2^ value of 0.985) between technical replicates (Additional file 5: Figure S5). Only RNA samples that were at a concentration ≥5 ng/μl were analysed in this assay.

The multiplex PCR assay was able to demonstrate statistically significant differences in the quality of the RNA released from the different age FFPE blocks (Fig. 4b). Across all the extraction kits, lower quality RNA was obtained from the blocks fixed between 2 and 3 years ago than those fixed <6 months ago. Furthermore, differences in the quality of the RNA released from different RNA extraction kits were also found to be statistically significant. The Maxwell 16 gave significantly higher quality RNA (lowest mean Cq value) than the three other extraction methods, although only 50% of samples were >5 ng/μl indicating a compromise in yield. The mean Cq value for the Maxwell 16 was 25.9, 2.4 Cq lower than the Arcturus Paradise Plus FFPE RNA isolation kit, 1.6 Cq lower than the RNeasy and 2.0 Cq lower than the Dual extraction kit. The Maxwell 16 is the only method which uses paramagnetic beads and the lack of mechanical force applied through centrifugation in this extraction technique may account for the higher quality of the RNA released. No significant differences in the mean Cq values between the two Qiagen extraction kits were seen. This data demonstrates the in house multiplex PCR assay is a much more sensitive approach, allowing multiple measures of RNA quality from minimal amounts of sample.

### Total amplifiable DNA recovery

A total of 120 DNA samples from 6 different FFPE cell line pellets were obtained using four different DNA extraction kits. 5–25 μm of tissue from each block was processed using each method. DNA concentration was quantified based on the amplification of a 41 bp DNA target from a conserved single copy DNA locus. Due to Qiagen kits not retaining products <100 bp, the DNA concentration was also quantified using amplication of a 129 bp DNA target.

**Fig. 5:**
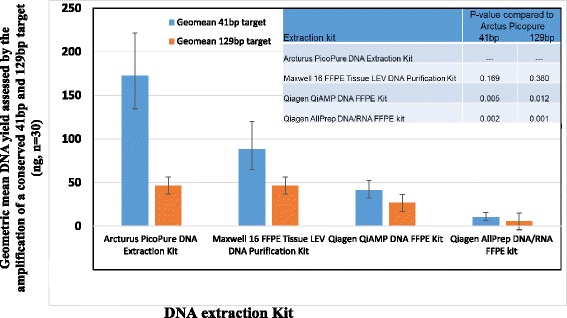
Comparison of DNA yields obtained using four off the shelf extraction kits assessed by amplification of a conversed 41 bp or 129 bp target: GeoMean DNA yield and associated standard error of geomean for the four extraction kits tested in this study, *n* = 30 for each method

All four kits successfully extracted amplifiable DNA from all samples as judged by a Cq value <35 for amplification of the 41 bp and 129 bp targets. PicoPure generated a superior geometric mean DNA yield of 172 ng in 41 bp assay in comparison to the Maxwell 16 (88 ng), QIAmp (40.9 ng) and AllPrep (10.1 ng) (Fig. 5/Table 1). However, the difference in yield seen between the Maxwell 16 and PicoPure were not significant (P-value 0.17). Single and dual DNA extraction kits from Qiagen gave significantly lower DNA yields than the other two kits, but were not significantly different from each other. The same conclusions were drawn from the 129 bp assay (Fig. 5). The PicoPure kit from Life Technologies is a single tube extraction method, thus avoiding the need to bind DNA to a solid interphase followed by laborious wash steps prior to eluting, all potentially resulting in lower DNA yields. This method comprises solely of a 24 h Proteinase K digestion which may account for the higher yields as longer incubation periods have been linked to the increased release of amplifiable DNA through more efficient reversal of formaldehyde induced cross linking [24]. Also, in contrast to the other kits tested, Proteinase K is stored in a lyophilised form in the PicoPure kit and is only reconstituted prior to extraction thus maintaining optimal enzyme activity. Furthermore, the PicoPure kit elutes DNA in a total volume of 150 μl, three times that of the other methods, which may also contribute to the superior yields (Table 1). Although the Maxwell 16 uses a magnetic particle movement automated extraction process, the total hands on time was greater than the manual PicoPure kit and therefore provided no advantage in terms of yield or efficiency.

Based on this data, the Arcturus PicoPure DNA extraction kit demonstrated superior performance in terms of DNA yield from FFPE tissue.

### Effect of tissue input on DNA concentration/yield using a fixed elution volume

Each DNA extraction method was tested using 5 μm, 10 μm, 15 μm, 20 μm and 25 μm of tissue from 6 different FFPE cancer cell line pellets. The relationship between DNA concentration, quantified by qPCR as above, and amount of tissue processed was investigated.

**Fig. 6:**
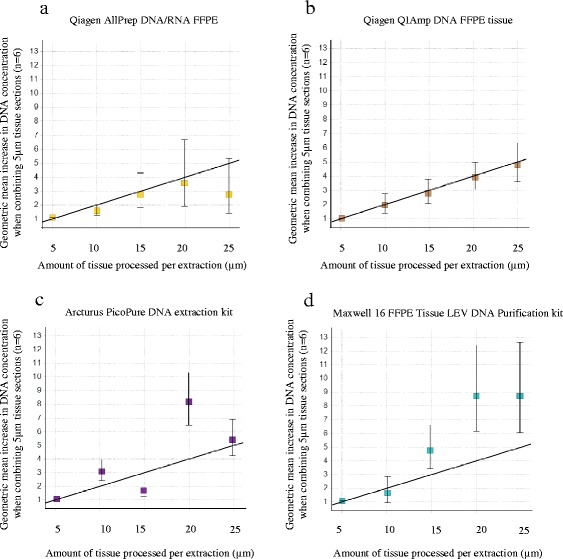
Investigating the relationship between DNA concentration and the input tissue amount: Geometric mean increase in DNA concentration across 6 different FFPE blocks when combining 5 μm sections in a linear fashion and associated standard error of geomean, line of Y = X/5 represents a linear relationship **a** Qiagen AllPrep DNA/RNA FFPE, **b** Qiagen QIAmp DNA FFPE tissue, **c** Arcturus PicoPure DNA extraction kit, **d** Maxwell 16 FFPE Tissue LEV DNA Purification Kit

Differences in extraction efficiencies were observed across the 4 kits tested. The Dual kit showed extraction efficiencies between 79 and 90% when processing up to 20 μm of tissue. The efficiency decreased by 55% when extracting >20 μm suggesting processing >20 μm of tissue in a single extraction would not be recommended due to potential loss of DNA (Fig. 6a). The QIAmp DNA FFPE extraction kit demonstrated a linear relationship between DNA concentration and the input amount of tissue (Fig. 6b). The Arcturus PicoPure DNA kit and the Maxwell 16 FFPE Tissue LEV DNA purification kit both produced extraction efficiencies >100% when processing up to 20 μm of tissue (Fig. 6c, d) indicating that for these kits using 5 μm sections is not optimal.

Overall results showed that extraction from 20 μm tissue sections gave optimal yields across all the kits (Fig. 2).

### DNA quality assessment

The quality of 120 DNA samples extracted from different age FFPE blocks using four different extraction kits was assessed using two qPCR assays targeting a 41 bp amplicon and a 129b amplicon from a conserved single copy DNA locus. The ratio between the concentration of the 129 bp amplicon and 41 bp amplicon (Q ratio) was used as a measure of DNA quality as fragmentation will impact the amplification of the longer DNA target, resulting in a lower ratio. However, running this assay in the recommended 20 μl reaction volume with sample duplicates, limits the ability to assess the quality of scarce clinical samples, so the assay was miniaturised to a 3.0 μl reaction volume.

**Fig. 7:**
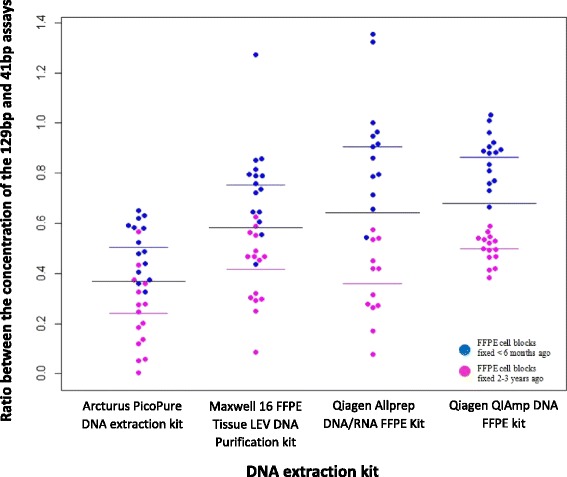
Comparison of the quality of DNA released using four off the shelf extraction kits: Relative quality of DNA samples calculated by normalising the concentration of 129 bp amplicon to the 41 bp amplicon using the KAPA Biosystems kit. from 6 different FFPE blocks either <6 months of age (*blue*) or between 2 and 3 years of age (*pink*), horizontal black lines represent the mean Cq for the entire sample population, with pink and blue lines for the two equal subgroups of FFPE blocks

All DNA extraction kits released higher quality DNA from FFPE blocks <6 months old with higher Q ratios (Fig. 7). The qPCR assay did show differences in the quality of the DNA released using different extraction kits. Significantly lower mean Q ratio of 0.37 was obtained with the Arcturus PicoPure DNA extraction kit compared to the other 3 DNA extraction methods, which were not significantly different to each other. The difference in mean ratio between the single and dual DNA extraction kits from Qiagen was a mere 0.1, showing that neither yield nor quality is compromised through dual extraction procedures.

Silica column and bead based extractions rely on the DNA binding ability of a solid phase. The columns supplied in the Qiagen single and dual DNA extraction kits are only able to bind DNA molecules ≥100 base pairs in length, resulting in the loss of the 41 bp fragment and selective enrichment of DNA fragments ≥100 base pairs. This leads to an over estimation of the ratio between the 129 bp target and the 41 bp target, so caution when analysing this data is required. In contrast, the Picopure DNA extraction kit is not reliant on the binding of DNA molecules to a solid phase and therefore all DNA fragments are retained, giving a more realistic representation of the quality of the DNA released from FFPE samples.

Data showed the miniaturised KAPA assay can be used to assess quantity and quality of DNA extracted without sacrificing large amounts of DNA (Fig. 7).

## Conclusions

Previous publications have compared the performance of manual FFPE extraction kits [16–18] and automated and manual kit counterparts [19–22], but different samples and methods for quantifying and assessing quality makes comparisons challenging. We have presented a comprehensive analysis of seven selected kits with a single sample set and compared directly platforms to assess quantity and quality of nucleic acids.

This study demonstrates that a number of factors influence the quantity and quality of nucleic acids obtained from FFPE samples, including the age of the samples, the extraction method, the amount of tissue processed and the elution volume. Following the manufacturer’s instructions, the data showed that no single protocol will consistently release high yields of high quality nucleic acids across all sample types.

Perhaps not surprisingly, our data shows that superior performance in high yields will often result in a compromise in concentration and/or quality. Therefore, downstream applications and nature of the samples to be tested should be given consideration up front before choosing the extraction method to apply.

Although FFPE cell line material was used in this study to remove sample heterogeneity, and supply issues, Qiagen RNeasy FFPE kit has been successfully utilised on clinical FFPE samples following this study [26]. Indicating that conclusions from this study are applicable to clinical FFPE tissue samples.

To potentially improve the performance of the kits tested in this study a wider validation study would need to be conducted, to test variables such as; method of deparaffinisation (deparaffinisation buffers vs. xylene), lysis volume and incubation time, column vs. cell lysate DNase digestion (for RNA isolation), and testing reagents such as Proteinase K from a lyophilised stock rather than a readymade solution.

This study successfully highlighted the key benefits of each extraction method tested, following the manufacturer’s protocols, with the aim to aid scientists in choosing a particular method dependent on individual goals. We provide a flow diagram to help question the properties that are most important to the success of studies with additional information around the most accurate and efficient methods to quantify and QC the extracted material (Fig. 2).

## Additional files

Additional file 1: Figure S1. Investigating the linearity of the multiplex PCR assay: relationship between cDNA concentration and mean Cq value for serial dilutions of a 10 ng/μl RNA sample ran in the multiplex PCR assay. (PDF 28 kb)

Additional file 2: Figure S2. Comparison of the Nanodrop and Qubit for RNA quantification: Correlation plot between RNA concentrations measured using the Nanodrop absorbance based assay and the Qubit fluorescence based assay, r^2^ represents the correlation coefficient, (a) Qiagen AllPrep DNA/RNA FFPE kit *n* = 30, (b) Qiagen RNeasy FFPE kit, *n* = 30, (c) Arcturus paradise plus FFPE RNA isolation kit, *n* = 25, (d) Maxwell 16 LEV RNA FFPE Purification kit, *n* = 30. (PDF 49 kb)

Additional file 3: Figure S3. Investigating the relationship between elution volume, RNA concentration and yield: Geometric mean RNA yield (orange bars) and geometric mean RNA concentration (black line) across four FFPE blocks when processing 5 μm of tissue using the RNeasy FFPE kit using varied elution volumes, error bars represent the standard error of geomean. (PDF 13 kb)

Additional file 4: Figure S4. Validation of the in house multiplex PCR assay: Correlation between the Cq values for each housekeeping gene for samples ran in both the single and multiplex PCR assay, r^2^ represents the correlation coefficient (PDF 24 kb)

Additional file 5: Figure S5. Assessing intra assay variation of the multiplex PCR assay: Correlation between the Cq values of technical replicates for each housekeeping gene for samples ran in the multiplex PCR assay, r^2^ represents the correlation coefficient. (PDF 34 kb)

Additional file 6: Table S1. Properties of the three primers and probes used in the in house multiplex PCR assay to asses RNA quality. Roche LightCycler480 PCR machine conditions: 1 pre incubation cycle at 95 °C for 10 min followed by 45 amplification cycles at 95 °C for 10 s, 60 °C for 30 s, 72 °C for 1 s before cooling to 40 °C for 30 s. (PDF 81 kb)

## Abbreviations

FFPE: Formalin fixed paraffin embedded

## References

[1] Kalmar A, Wichmann B, Galamb O, Spisák S, Tóth K, Leiszter K, Tulassay Z, Molnár B (2013). Gene expression analysis of normal and colorectal cancer tissue samples from fresh frozen and matched formalin-fixed, paraffin-embedded (FFPE) specimens after manual and automated RNA isolation. Methods.

[2] Northcott P, Shih D, Remke M, Cho YJ, Kool M, Hawkins C, Eberhart C, Dubuc A, Guettouche T, Cardentey Y, Bouffet E, Pomeroy S, Marra M, Malkin D, Rutka J, Korshunov A, Pfister S, Taylor M (2012). Rapid, reliable, and reproducible molecular sub-grouping of clinical medulloblastoma samples. Acta Neuropathol.

[3] Rahimov F, King OD, Leung DG, Bibat GM, Emerson CP, Kunkel LM, Wagner KR (2012). Transcriptional profiling in facioscapulohumeral muscular dystrophy to identify candidate biomarkers. Proc Natl Acad Sci U S A.

[4] Sun Z, Asmann YW, Kalari KR, Bot B, Eckel-Passow JE, Baker TR, Carr JM, Khrebtukova I, Luo S, Zhang L, Schroth G, Perez EA, Thompson EA (2011). Integrated analysis of gene expression, CpG island methylation, and gene copy number in breast cancer cells by deep sequencing. PLoS One.

[5] Valleron W, Ysebaert L, Berquet L, Fataccioli V, Quelen C, Martin A, Parrens M, Lamant L, de Leval L, Gisselbrecht C, Gaulard P, Brousset P (2012). Small nucleolar RNA expression profiling identifies potential prognostic markers in peripheral T-cell lymphoma. Blood.

[6] Levy MA, Lovly CM, Pao W (2012). Translating genomic information into clinical medicine: lung cancer as a paradigm. Genome Res.

[7] Mayeux R (2004). Biomarkers: potential uses and limitations. NeuroRx.

[8] Simon R (2011). Genomic biomarkers in predictive medicine: an interim analysis. EMBO Mol Med.

[9] Asslaber M, Zatloukal K (2007). Biobanks: transnational, European and global networks. Brief Funct Genomic Proteomic.

[10] Moore H, Compton C, Alper J, Vaught J (2009). 2009 Biospecimen research network symposium: advancing cancer research through biospecimen science. Cancer Res.

[11] Srinivasan M, Sedmak D, Jewell S (2002). Effect of fixatives and tissue processing on the content and integrity of nucleic acids. Am J Pathol.

[12] Florell SR, Coffin CM, Holden JA, Zimmermann JW, Gerwels JW, Summers BK, Jones DA, Leachman SA (2001). Preservation of RNA for functional genomic studies: a multidisciplinary tumor bank protocol. Mod Pathol.

[13] Masuda N, Ohnishi T, Kawamoto S, Monden M, Okubo K (1999). Analysis of chemical modification of RNA from formalin-fixed samples and optimization of molecular biology applications for such samples. Nucleic Acids Res.

[14] McGhee JD, von Hippel PH (1977). Formaldehyde as a probe of DNA structure. r. Mechanism of the initial reaction of Formaldehyde with DNA. Biochemistry.

[15] Williams C, Pontén F, Moberg C, Söderkvist P, Uhlén M, Pontén J, Sitbon G, Lundeberg J (1999). A high frequency of sequence alterations is due to formalin fixation of archival specimens. Am J Pathol.

[16] Dedhia P, Tarale S, Dhongde G, Khadapkar R, Das B (2007). Evaluation of DNA extraction methods and real time PCR optimization on formalin-fixed paraffin-embedded tissues. Asian Pac J Cancer Prev.

[17] Huang WY, Sheehy TM, Moore LE, Hsing AW, Purdue MP (2010). Simultaneous recovery of DNA and RNA from formalin-fixed paraffin-embedded tissue and application in epidemiologic studies. Cancer Epidemiol Biomarkers Prev.

[18] Okello JB, Zurek J, Devault AM, Kuch M, Okwi AL, Sewankambo NK, Bimenya GS, Poinar D, Poinar HN (2010). Comparison of methods in the recovery of nucleic acids from archival formalin-fixed paraffin-embedded autopsy tissues. Anal Biochem.

[19] Bohmann K, Hennig G, Rogel U, Poremba C, Mueller BM, Fritz P, Stoerkel S, Schaefer KL (2009). RNA extraction from archival formalin-fixed paraffin-embedded tissue: a comparison of manual, semiautomated, and fully automated purification methods. Clin Chem.

[20] Hennig G, Gehrmann M, Stropp U, Brauch H, Fritz P, Eichelbaum M, Schwab M, Schroth W (2010). Automated extraction of DNA and RNA from a single formalin-fixed paraffin-embedded tissue section for analysis of both single-nucleotide polymorphisms and mRNA expression. Clin Chem.

[21] Khokhar SK, Mitui M, Leos NK, Rogers BB, Park JY (2012). Evaluation of Maxwell(R) 16 for automated DNA extraction from whole blood and formalin-fixed paraffin embedded (FFPE) tissue. Clin Chem Lab Med.

[22] Sam SS, Lebel KA, Bissaillon CL, Tafe LJ, Tsongalis GJ, Lefferts JA (2012). Automation of genomic DNA isolation from formalin-fixed, paraffin-embedded tissues. Pathol Res Pract.

[23] Ribeiro-Silva A, Zhang H, Jeffrey SS (2007). RNA extraction from ten year old formalin-fixed paraffin-embedded breast cancer samples: a comparison of column purification and magnetic bead-based technologies. BMC Mol Biol.

[24] Gilbert MT, Haselkorn T, Bunce M, Sanchez JJ, Lucas SB, Jewell LD, Van Marck E, Worobey M (2007). The isolation of nucleic acids from fixed, paraffin-embedded tissues-which methods are useful when?. PLoS One.

[25] Stanta G, Schneider C (1991). RNA extracted from paraffin-embedded human tissues is amenable to analysis by PCR amplification. Biotechniques.

[26] Veldman-Jones MH, Brant R, Rooney C, Geh C, Emery H, Harbron CG, Wappett M, Sharpe A, Dymond M, Barrett JC, Harrington EA, Marshall G (2015). Evaluating robustness and sensitivity of the NanoString technologies nCounter platform to enable multiplexed gene expression analysis of clinical samples. Cancer Res.

