# Supplementary figures and images for: Nucleic acid extraction from formalin-fixed paraffin-embedded cancer cell line samples: a trade off between quantity and quality?
Source: BMC Clin Pathol. 2016 Nov 14;16:17. doi: 10.1186/s12907-016-0039-3 (PMC5477763; doi:10.1186/s12907-016-0039-3)

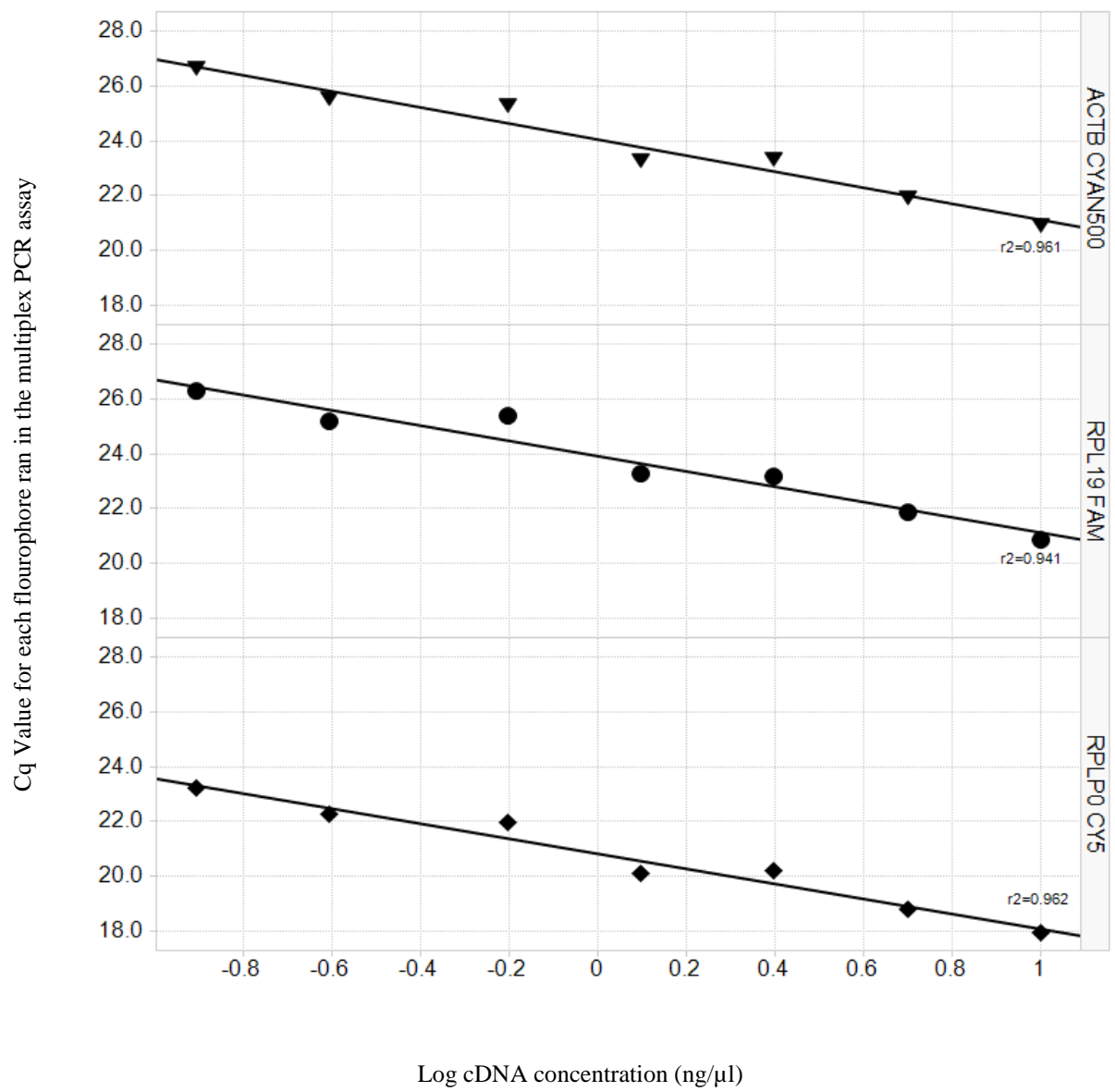

Supplement: Supplementary file 1 — Investigating the linearity of the multiplex PCR assay: relationship between cDNA concentration and mean Cq value for serial dilutions of a 10 ng/μl RNA sample ran in the multiplex PCR assay. (PDF 28 kb) [file 12907_2016_39_MOESM1_ESM.pdf]

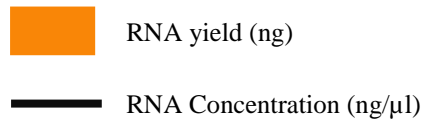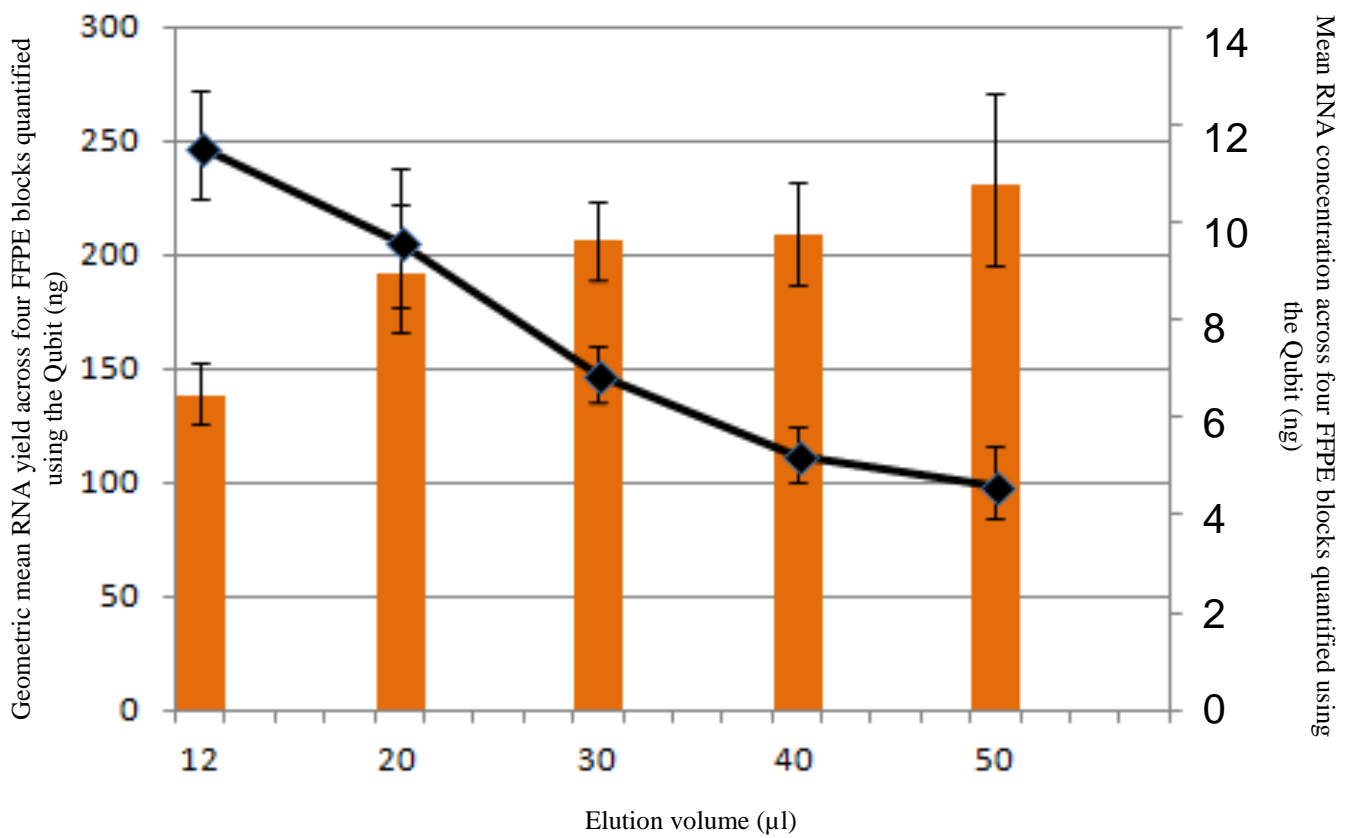

Supplement: Supplementary file 3 — Investigating the relationship between elution volume, RNA concentration and yield: Geometric mean RNA yield (orange bars) and geometric mean RNA concentration (black line) across four FFPE blocks when processing 5 μm of tissue using the RNeasy FFPE kit using varied elution volumes, error bars represent the standard error of geomean. (PDF 13 kb) [file 12907_2016_39_MOESM3_ESM.pdf]

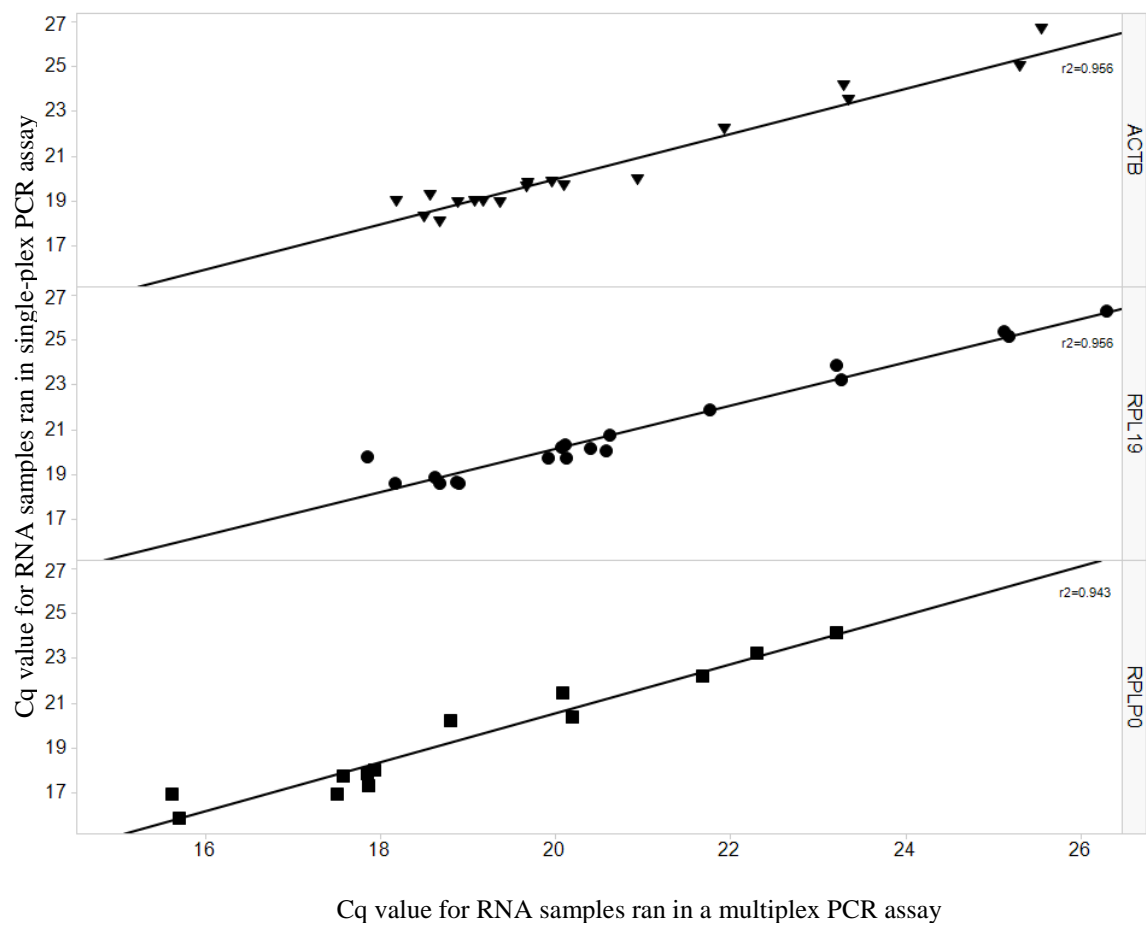

Supplement: Supplementary file 4 — Validation of the in house multiplex PCR assay: Correlation between the Cq values for each housekeeping gene for samples ran in both the single and multiplex PCR assay, r2 represents the correlation coefficient (PDF 24 kb) [file 12907_2016_39_MOESM4_ESM.pdf]

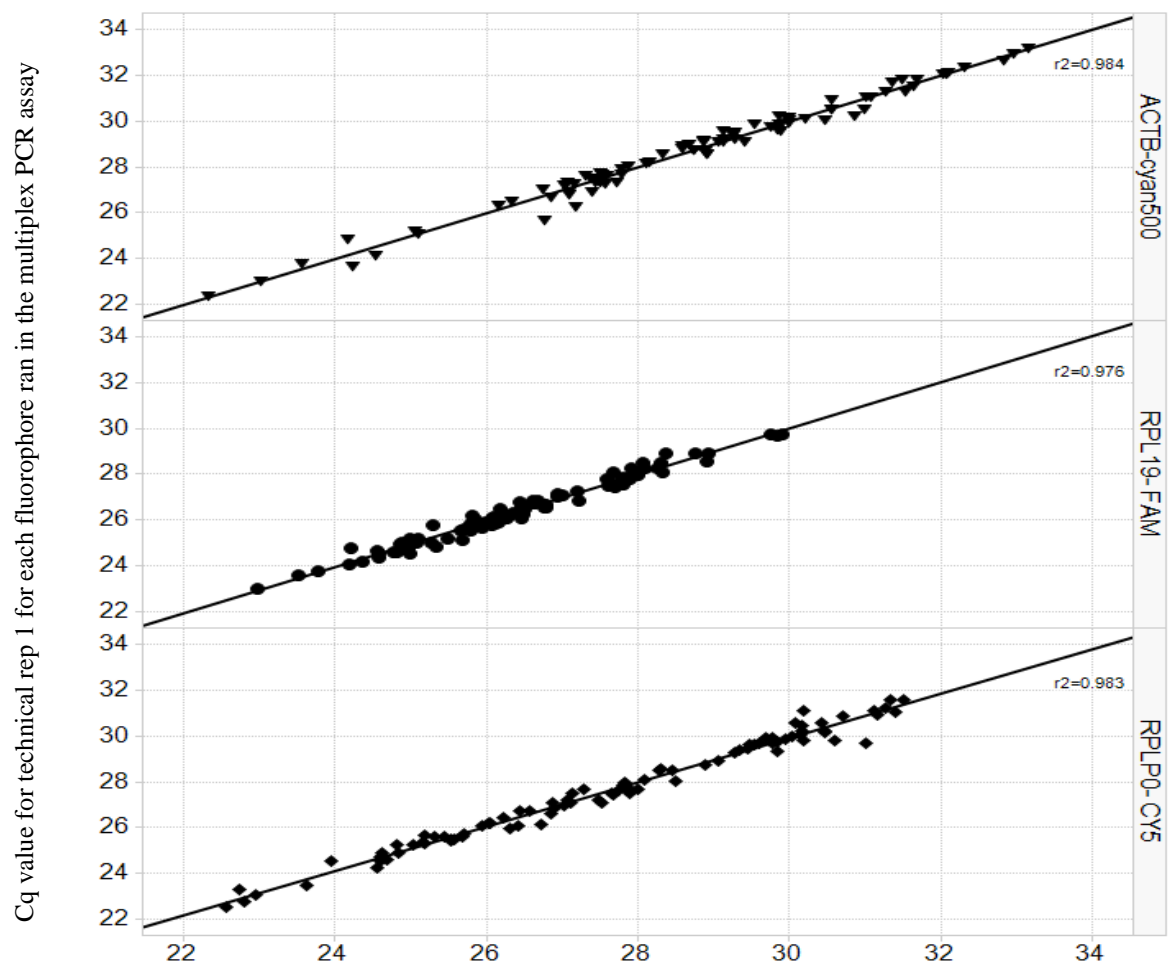

Cq value for technical rep 2 for each fluorophore ran in the multiplex PCR assay

Supplement: Supplementary file 5 — Assessing intra assay variation of the multiplex PCR assay: Correlation between the Cq values of technical replicates for each housekeeping gene for samples ran in the multiplex PCR assay, r2 represents the correlation coefficient. (PDF 34 kb) [file 12907_2016_39_MOESM5_ESM.pdf]
