# Supplementary material for: Nucleic acid extraction from formalin-fixed paraffin-embedded cancer cell line samples: a trade off between quantity and quality?
Source: BMC Clin Pathol. 2016 Nov 14;16:17. doi: 10.1186/s12907-016-0039-3 (PMC5477763; doi:10.1186/s12907-016-0039-3)

1a. Qiagen AllPrep DNA/RNA FFPE

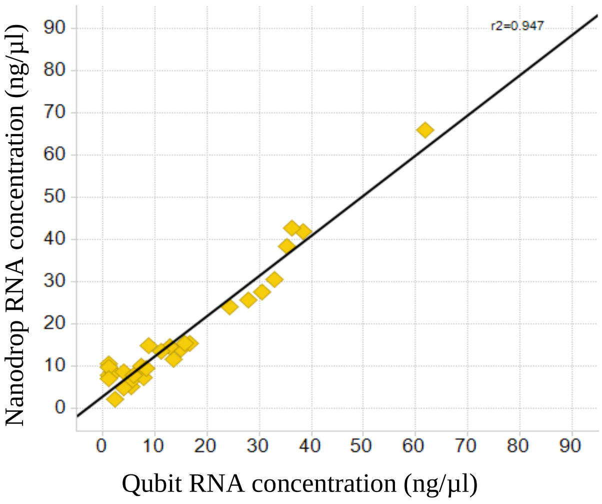

1b. Qiagen RNeasy FFPE Kit

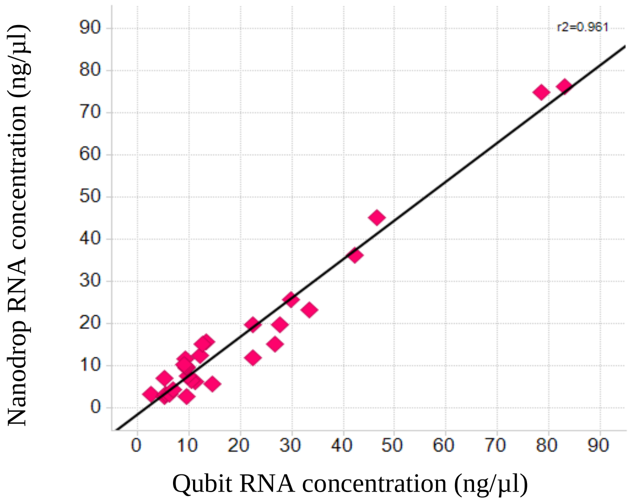

1c. Arcturus paradise plus FFPE RNA isolation kit

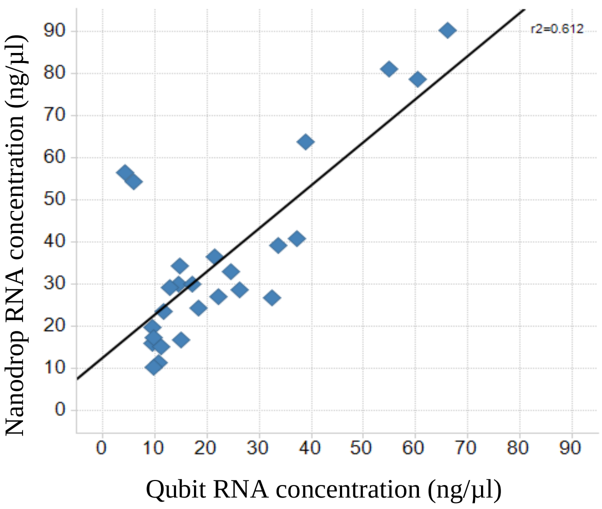

1d. Maxwell 16 LEV RNA FFPE Purification kit

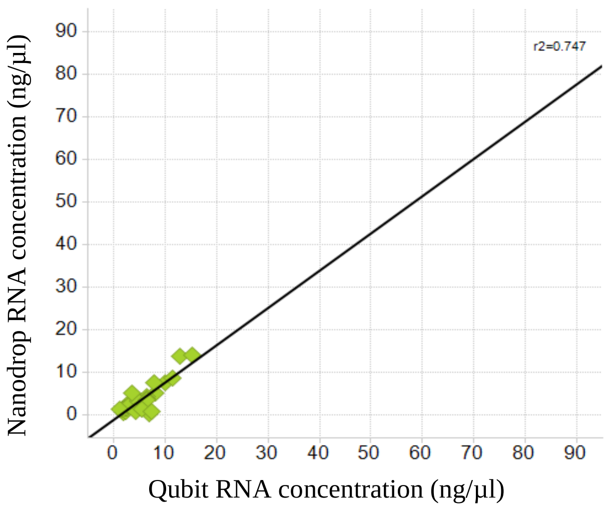

Supplement: Supplementary file 2 — Comparison of the Nanodrop and Qubit for RNA quantification: Correlation plot between RNA concentrations measured using the Nanodrop absorbance based assay and the Qubit fluorescence based assay, r2 represents the correlation coefficient, (a) Qiagen AllPrep DNA/RNA FFPE kit n = 30, (b) Qiagen RNeasy FFPE kit, n = 30, (c) Arcturus paradise plus FFPE RNA isolation kit, n = 25, (d) Maxwell 16 LEV RNA FFPE Purification kit, n = 30. (PDF 49 kb) [file 12907_2016_39_MOESM2_ESM.pdf]
