# Supplementary material for: Nucleic acid extraction from formalin-fixed paraffin-embedded cancer cell line samples: a trade off between quantity and quality?
Source: BMC Clin Pathol. 2016 Nov 14;16:17. doi: 10.1186/s12907-016-0039-3 (PMC5477763; doi:10.1186/s12907-016-0039-3)
Supplement: Supplementary file 6 — Properties of the three primers and probes used in the in house multiplex PCR assay to asses RNA quality. Roche LightCycler480 PCR machine conditions: 1 pre incubation cycle at 95 °C for 10 min followed by 45 amplification cycles at 95 °C for 10 s, 60 °C for 30 s, 72 °C for 1 s before cooling to 40 °C for 30 s. (PDF 81 kb) [file 12907_2016_39_MOESM6_ESM.pdf]

Supplemental Table S1. Properties of the three primers and probes used in the in house multiplex PCR assay to asses RNA quality. Roche LightCycler480 PCR machine conditions:

1 pre incubation cycle at 95°C for 10 minutes followed by 45 amplification cycles at 95°C for 10 seconds, 60°C for 30 seconds, 72°C for 1 second before cooling to 40°C for 30 seconds.

Target Gene Symbol	RPLP0	RPL19	ACTB
Sequence accession number	NM_001002	NM_000981	NM_001101
Amplicon Length (base pairs)	Chromosome 12	Chromosome 17	Chromosome 7
Amplicon Length	144bp	68bp	137bp
Primer Location	Exon 2 and 3<br>394 – 537 bases	Exon 3, 4<br>418 – 485 bases	Exon 1,2,3,4,5<br>855 - 991 bases
Splice Variants Targeted	Transcript 1, 2, 4	Transcript 1,2,3	Transcript 1,2,3,4,7,8,9
Forwards Primer Sequence 5'3'	AATGTTTCAT TGTGGGAGCA	AGGAGAATGA GGATTTTGCG	TGGACTTCG AGCAAGAGTG
Reverse Primer Sequence 5'3'	CTGGGTTGT TTTCCAGGTG	ATGTGGCGA TCGATCTTCTT	GAAGGAAGGC TGGAAGAGGTG
Probe Sequence 5'3'	CTTCGCGGG AAGGCTCTGGT	CCGGCTGCTCA GAAGATACCGTG	CGGCTGCTTC CAGCTCCTCC
Modifications	5' Cy5 3' BHQ-2	5' 6-FAM 3' BHQ-1	5' Cyan500 3' BHQ-2
Final Primer Concentration	10µM	10µM	10µM
Final Probe Concentration	5µM	5µM	5µM
Purification Method	Primers: RP Cartridge, Probe: RPL HCLC	Primers: RP Cartridge, Probe: RPL HCLC	Primers: RP Cartridge, Probe: RPL HCLC
